# Does a Dual Plane with Gland Suspension Really Improve Outcomes after Breast Augmentation?

**DOI:** 10.1097/GOX.0000000000002247

**Published:** 2019-04-25

**Authors:** Eric Swanson

**Affiliations:** From the Swanson Center, Leawood, Kans.

## Sir:

Andjelkov et al.^1^ claim that their breast suspension maneuver, in addition to a dual plane breast augmentation, improves breast augmentation outcomes. The authors base their conclusion on a reduced reintervention (reoperation) rate. Andjelkov et al. compare a group of women treated with their suspension/dual plane method versus a historical control group. The authors believe that their method avoids the need for a mastopexy in all women, except those with severe breast ptosis.^1^ The authors did include a 3-dimensional measurement device but used it in only 4 patients. Reoperation rates are not known to be reliable because they are affected by a host of variables that do not necessarily reflect the quality of the surgical result.^2^

**Fig. 1.**
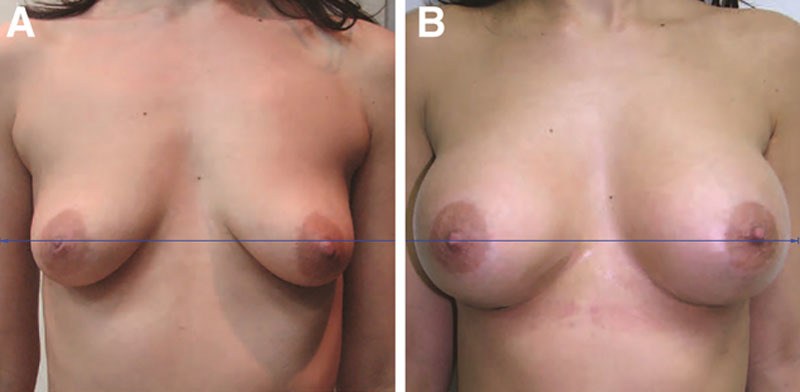
These frontal photographs depict a 31-year-old woman before (A) and 1 year after (B) a breast augmentation using the authors’ method. The original postoperative photograph was enlarged 15% compared with the preoperative photograph and positioned 5 cm higher. After correction for size, position, and a slight (2°) tilt, using the Canfield 7.4.1 Mirror imaging software (Canfield Scientific, Fairfield, N.J.), there appears to be very little elevation of the nipple. The lower pole level has dropped. (Adapted from Fig. 5, Andjelkov et al.^1^)

The video simply shows placement of a single absorbable suture between the breast tissue and pectoralis fascia superiorly in the prepectoral pocket.^1^ It is not clear that this maneuver is effective in elevating the breast mound. If it did fix the breast tissue to the muscle, distortion on muscle movement might be a problem.^3^ Importantly, the illustrations are not standardized. The postoperative photographs are magnified and the torso is positioned much higher after surgery, making it appear that the breasts have been substantially elevated on the chest wall. This difference is easy to spot by simply lining up pigmented nevi. When the photographs are corrected for tilt, magnification, and position, the degree of elevation appears much more modest (Fig. 1).

Tebbetts^4^ claims that a partial prepectoral dissection, in addition to a subpectoral dissection, improves breast shape in women with glandular ptotis or constricted lower poles and elevates the nipple. However, this claim has never been substantiated with measurements. This maneuver does not really create a separate plane (the implant occupies only 1 plane)^5^ but does release the inferior margin of the pectoralis. Any benefit from this release is unclear. When compared with a traditional subpectoral breast augmentation, there is no advantage in measurable breast parameters, such as upper pole projection, breast projection, or lower pole level using the Type 3 modification.^5^

Suture suspension methods have long held an intuitive appeal to plastic surgeons, but are ineffective.^3,6^ It is also intuitive to believe that a breast implant takes up the slack and elevates the breast and nipple.^7^ However, plastic surgeons who have rigorously examined the breasts after augmentation using measurements find no significant nipple elevation.^3,7^ On the contrary, the lower pole level descends after breast augmentation, as does the inframammary fold.^3,7^ Breast augmentation is not a substitute for a mastopexy.^7^ Patients who lift their breasts up by the cups of both hands to demonstrate what they want are best served with a vertical augmentation/mastopexy.^7^ The minus/plus concept^8^ makes use of breast implants to provide upper pole fullness, and a vertical mastopexy to treat glandular ptosis and effectively elevate the breast.^7^ As evidenced by the illustrations in this study, photographic standardization is mandatory. Without an objective evaluation, it is easy to be convinced that one’s technique provides a superior result when the measurements suggest otherwise.
